# Comparing glycemic traits in defining diabetes among rural Chinese older adults

**DOI:** 10.1371/journal.pone.0296694

**Published:** 2024-01-25

**Authors:** Pin Wang, Yuanjing Li, Mingqi Wang, Lin Song, Yi Dong, Xiaolei Han, Jaakko Tuomilehto, Yongxiang Wang, Yifeng Du, Chengxuan Qiu

**Affiliations:** 1 Department of Neurology, Shandong Provincial Hospital, Jinan, Shandong Province, P.R. China; 2 Cheeloo College of Medicine, Shandong University, Jinan, Shandong Province, P.R. China; 3 Department of Neurobiology, Aging Research Center, Care Sciences and Society, Karolinska Institutet-Stockholm University, Stockholm, Sweden; 4 Public Health Promotion Unit, Finnish Institute for Health and Welfare, Helsinki, Finland; 5 Department of Public Health, University of Helsinki, Helsinki, Finland; 6 Saudi Diabetes Research Group, King Abdulaziz University, Jeddah, Saudi Arabia; 7 Department of International Health, National School of Public Health, Instituto de Salud Carlos III. Madrid, Spain; 8 Qingdao Endocrinology and Diabetes Hospital, Qingdao, Shandong Province, P.R. China; Muhimbili University of Health and Allied Sciences School of Medicine, UNITED REPUBLIC OF TANZANIA

## Abstract

**Background:**

We sought to identify the optimal cut-off of glycated hemoglobin (HbA1c) for defining diabetes and to assess the agreements of fasting plasma glucose (FPG), fasting serum glucose (FSG), and HbA1c in defining diabetes among rural older adults in China.

**Methods:**

This population-based cross-sectional study included 3547 participants (age ≥61 years, 57.8% women) from the Multidomain Interventions to Delay Dementia and Disability in Rural China from 2018–2019; of these, 3122 had no previously diagnosed diabetes. We identified the optimal cut-off of HbA1c against FPG ≥7.0 mmol/L for defining diabetes by using receiver operating characteristic curve and Youden index. The agreements of FPG, FSG, and HbA1c in defining diabetes were assessed using kappa statistics.

**Results:**

Among participants without previously diagnosed diabetes (n = 3122), the optimal HbA1c cut-off for defining diabetes was 6.5% (48 mmol/mol), with the sensitivity of 88.9%, specificity of 93.7%, and Youden index of 0.825. The correlation coefficients were 0.845 between FPG and FSG, 0.574 between FPG and HbA1c, and 0.529 between FSG and HbA1c in the total sample (n = 3547). The kappa statistic for defining diabetes was 0.962 between FSG and FPG, and 0.812 between HbA1c and FPG.

**Conclusions:**

The optimal cut-off of HbA1c for diagnosing diabetes against FPG >7.0 mmol/L is ≥6.5% in Chinese rural-dwelling older adults. The agreement in defining diabetes using FPG, FSG, and HbA1c is nearly perfect. These results have relevant implications for diabetes research and clinical practice among older adults in China.

**Clinical trial registration:**

The protocol of MIND-China was registered in the Chinese Clinical Trial Registry (ChiCTR, www.chictr.org.cn; registration no.: ChiCTR1800017758).

## 1. Introduction

In 2018–2019, the nationwide survey indicated that diabetes affected 12.4% of the adult population in China, with a nearly five-time increase from 2.5% in 1994 [1, 2]. China has the world highest number of people with diabetes, with 129.8 million people being affected by diabetes in mainland China [3]. In addition, the disease and economic burden of diabetes in China was projected to significantly increase in 2020–2030, and the increase would exceed that of gross domestic product [4]. Because type 2 diabetes is asymptomatic in its early stages, a considerable proportion of people with diabetes may not receive timely diagnosis until some symptoms of the disease or its complications appear. Thus, simple and reliable tests for hyperglycemia are crucial for early identification of diabetes and timely initiation of appropriate treatment.

Accordingly, several methods have also been proposed to assess glycemic status in diagnosing diabetes, such as the fasting plasma glucose (FPG), two-hour or one-hour post-challenge plasma glucose in the oral glucose tolerance test (2-hPG, 1-hPG), fasting serum glucose (FSG), whole-blood fasting and 2-h glucose, and glycated hemoglobin (HbA1c) [5–7]. Among them, the 2-hPG is a somewhat cumbersome test that requires several conditions in application (e.g., stable diet, an overnight fast, oral 75g glucose load, and repeated collection of blood samples after two hours). Thus, it is not always feasible to use 2-hPG in the screening and diagnosis of diabetes among older adults in the general population settings [8]. In 2003, the American Diabetes Association (ADA) proposed to use FPG as the primary method for diagnosing diabetes [9], thereafter FPG has been widely used in the diagnosis of diabetes in large-scale population settings [10, 11]. In addition, FSG could be measured simultaneously with other serum biochemical markers (e.g., lipids) from the same blood specimen, which is convenient and cost-effective compared with the measurement of FPG that requires a special tube for the collection of blood samples. Thus, FSG has been widely used in studies of the general population settings as a substitute of FPG for the diagnosis of diabetes [6, 12]. In terms of the correlation of these glycemic measures for diagnosing diabetes, data from the Finnish Diabetes Prevention Study found no difference between FPG and FSG in freshly collected blood samples [13]. Whereas, when there is a time delay in the process of measuring the serum glucose level, the glucose concentrations in serum are slightly lower than in fluoride plasma, but higher than in EDTA (ethylene diamine tetra acetic acid) plasma [14]. Therefore, the correlation and agreement between FPG and FSG remain to be carefully evaluated. In addition, HbA1c has been recommended by ADA and WHO as an alternative for diagnosing diabetes (cut-off ≥6.5% or >48 mmol/mol), which could reflect chronic hyperglycemia with low time-dependent variability and no requirement of fasting [15, 16]. However, very few population-based studies have estimated or validated the optimal cut-off for HbA1c among elderly in middle- and low-income countries, and due to its high cost, it is not feasible in many low-income settings.

Thus, in this population-based cross-sectional study, we aimed to identify the optimal cut-off of HbA1c against FPG in diagnosing diabetes and further to assess the agreement of FPG, FSG, and HbA1c in defining diabetes among rural-dwelling Chinese older adults.

## 2. Materials and methods

### 2.1 Study participants

This study is a population-based study. The study sample was derived from participants in the survey of recruitments for the cluster-randomized controlled Multidomain Interventions to Delay Dementia and Disability in Rural China (MIND-China) study [17]. In brief, in March-September 2018, the baseline examination of MIND-China targeted 5765 people who were aged 60 years and above and living in the 52 villages of Yanlou Town 52 villages, Yanggu County, western Shandong Province, China. In April-May 2019, a total of 3956 individuals who undertook the 2018 examination were invited for the further recruitments of participation in the MIND-China interventions. We did not invite people who were aged ≥80 years because by the study design of MIND-China, the intervention phases only targeted persons who were aged 60–79 years in 2018, is that 61–80 years in 2019. Out of the 3956 participants, 139 were excluded due to severe mental disorders, and severe problems with vision, hearing, or language, and the remaining 3817 participants underwent annual health check-up, a part of health care program for older adults provided by local government. Of these, we further excluded those with missing laboratory blood glucose tests (n = 242) or missing information on self-reported history of diabetes (n = 28), leaving 3547 persons (89.7% of the 3956 eligible persons) who were aged 61–80 years and free of dementia and disability in 2019 for the current analyses.

All parts of the MIND-China Project were approved by the Ethical Committee of Shandong Provincial Hospital affiliated to Shandong University, Jinan, Shandong. Prior to the survey, written informed consent was obtained from all participants, or in the case of persons with severe cognitive impairment, from proxies (usually a family member).

### 2.2 Data collection and assessments

In March-September 2018, trained medical staff for the survey collected data through face-to-face interviews, clinical examination, and testing following a structural questionnaire. These data included demographics (i.e., age, sex, and education), lifestyles (e.g., smoking, alcohol intake, and physical activity), health history (e.g., mental disorders, diabetes, hypertension, stroke, and coronary artery disease), and medical treatment.

In April-May 2019, venous blood specimens were drawn into three separate vacuum tubes after an overnight fast. One was filled with coagulant and allowed to clot. After centrifugation at 3000 rpm for 10 minutes (Jiawen JM-1048, Anhui), FSG and serum total cholesterol were measured along with other biochemical markers within two hours at clinical laboratory of the local town hospital using an automatic Biochemical Analyzer (CS-600B, DIRUI Corporation, Changchun, China), in which the glucose oxidase method was used. On the same day of blood specimen collection, other two tubes of blood samples were stored at 4°C and were transported to a laboratory at the Jinan KingMed Medical Test Center (certified by the National Glycohemoglobin Standardization Program) for analyses of FPG and HbA1c. The blood sample in a tube with sodium fluoride was centrifuged at 3000 rpm for 10 min (Jiawen JM-1048, Anhui) and FPG was analyzed in the same day of blood sample collection using the hexokinase method on an automatic analyzer (Beckman Coulter AU5821, USA). Then, another blood sample, which was drawn into a vacuum tube with Ethylene diamine tetra acetic acid, was used to measure HbA1c within two days at the laboratory of Jinan KingMed Medical Test Center by high-performance liquid chromatography using automatic hemoglobin testing system (Huizhong MQ6000, Shanghai, China).

We classified education as illiterate (no formal schooling), primary school, and middle school or above, and physical activity as at least weekly versus less than weekly activity. We categorized smoking and alcohol intake as never, former, and current. Weight and height were measured in light cloths without shoes. Body mass index was calculated as weight (kg) divided by height squared (m^2^). After a 5-min rest, sitting arterial blood pressure and heart rate were measured on the right upper arm of the person in a seated position using an electronic blood pressure monitor (Omron HEM-7127J; Omron Corporation, Kyoto, Japan).

Coronary heart disease and clinical stroke were ascertained according to self-reported medical history and clinical and neurological examinations during the annual health check-ups. We defined high serum cholesterol as total cholesterol ≥6.22 mmol/L, or having received treatment for high cholesterol and hypertension as systolic pressure ≥140 mmHg, or diastolic pressure ≥90 mmHg, or current use of antihypertensive drugs[12]. Diabetes was considered to be present if the participant had either self-reported physician diagnosis of diabetes or current use of glucose-lowering drugs or high glycemia in one of the three glycemic measurements [18]. We assessed optimal cut-off of HbA1c and compared three different methods for defining diabetes against FPG ≥7.0 mmol/L, because the 2-hPG is not feasible in large-scale general population setting of older adults. Participants who were either treated with glucose-lowering drugs or recorded to have diabetes in the health check-up record system were defined as having previously diagnosed diabetes. All relevant information of the participants was collected by the well-trained medical staff.

### 2.3 Statistical analysis

Characteristics of the study participants by diabetes status were compared using t-test for continuous variables and χ^2^ test for categorical variables. Missing values of continuous variables are filled by average values, and that of categorical variables are filled by mode. We examined the optimal cut-off of HbA1c in diagnosing diabetes only among people without previous diagnosis of diabetes, given that the level of HbA1c might be influenced by lifestyle modifications or use of glucose-lowering drugs among people with previous diagnosis of diabetes. We assessed optimal cut-off of HbA1c for defining diabetes against FPG ≥7.0 mmol/L by using the receiver operating characteristic (ROC) curve in participants without previous history of diabetes and assessed the sensitivity and specificity of HbA1c in diagnosing diabetes. Next, in the total sample, we estimated the parameters of diagnostic accuracy (i.e., Youden index, positive and negative likelihood ratios, and positive and negative predictive values) according to 1–3 SDs above mean value of HbA1c in participants with normal glucose tolerance, respectively. The identified optimal cut-off was used for subsequent analyses. We employed Spearman’s correlation coefficient to assess the correlation among FPG, FSG, and HbA1c. We used kappa statistics to evaluate the diagnostic agreement of different methods for defining diabetes in the total sample. We calculated the kappa using the formular: (P0-Pe)/(1-Pe) [19]. IBM SPSS Statistics 25.0 for Windows (IBM Corp., Armonk, NY) was used for all analyses.

## 3. Results

### 3.1 Characteristics of study participants (n = 3547)

The average age of the 3547 participants was 69.5 years (SD, 4.2), and 57.8% were female. When diabetes was defined by fasting plasma glucose ≥7.0 mmol/L, use of glucose-lowering drug treatment, or medical record of diabetes diagnosis, participants with diabetes reached 506. Compared with participants without diabetes (n = 3041), those with diabetes were slightly younger, more likely to be female, to have hypertension, history of stroke and coronary artery disease, and higher levels of BMI, and less likely to smoke or drink alcohol (p<0.001, Table 1).

**Table 1:** Characteristics of study participates according to diabetes status (n = 3547).

Characteristics	Study sample	Diabetes status^*^		
	(n = 3547)	No (n = 3041)	Yes (n = 506)	P-value
Age, years	69.5 (4.2)	69.6 (4.2)	68.8 (4.0)	<0.001
Female, n (%)	2049 (57.8)	1705 (56.1)	344 (68.0)	<0.001
Educational level, n (%)				0.201
Illiterate	1323 (37.3)	1119 (36.8)	204 (40.3)	
Primary school	1570 (44.3)	1364 (44.9)	206 (40.7)	
Middle school and above	654 (18.4)	558 (18.3)	96 (19.0)	
Physical activity, n (%)				0.408
Less than weekly	2948 (83.1)	2521 (82.9)	427 (84.4)	
At least weekly	599 (16.9)	520 (17.1)	79 (15.6)	
Current smoking, n (%)	790 (22.3)	726 (23.9)	64 (12.6)	<0.001
Current alcohol intake, n (%)	1073 (30.3)	956 (31.4)	117 (23.1)	<0.001
Body mass index, kg/m^2^	25.0 (3.6)	24.8 (3.6)	26.0 (3.5)	<0.001
Hypertension, n (%)	2585 (72.9)	2179 (71.7)	406 (80.2)	<0.001
High serum cholesterol level, n (%)	359 (10.1)	302 (9.9)	57 (11.3)	0.357
Stroke, n (%)	473 (13.3)	380 (12.5)	93 (18.4)	<0.001
Coronary artery disease, n (%)	672 (18.9)	542 (17.8)	130 (25.7)	<0.001
Fasting plasma glucose, mmol/l	5.4 (1.4)	5.1 (0.5)	7.7 (2.4)	<0.001
Fasting serum glucose, mmol/l	5.2 (1.4)	4.9 (0.7)	7.4 (2.4)	<0.001
Glycated hemoglobin A1c, %; mmol/mol	6.1 (1.0); 42.6 (10.9)	5.8 (0.4); 39.7 (4.7)	7.7 (1.6); 60.5 (18.0)	<0.001

Data were mean (SD), unless otherwise specified.

^*^ Diabetes was defined by fasting plasma glucose ≥7.0 mmol/L, use of glucose-lowering drug treatment, or medical record of diabetes diagnosis.

The two groups did not differ significantly in the distributions of the educational level, physical activity, and high serum cholesterol.

### 3.2 Optimal HbA1c cut-off for the diagnosis of diabetes

**Fig 1:**
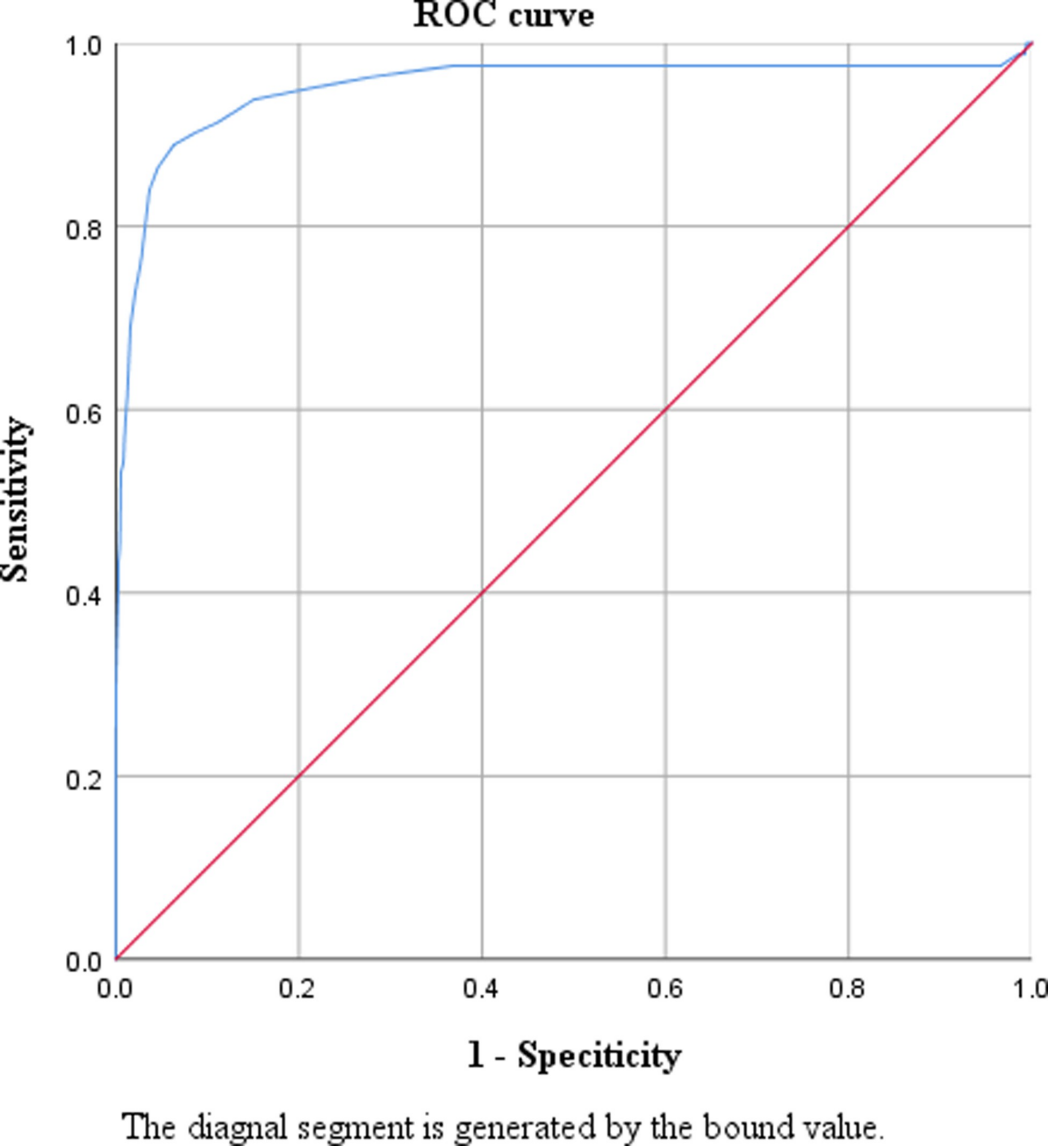
The receiver operating characteristics curve for diagnosing diabetes using hemoglobin A1c ≥6.5% compared with FPG >7.0 mmol/L among people without previously diagnosed diabetes (n = 3122). The optimal cut-off value of hemoglobin A1c was ≥6.5% for diagnosing diabetes, with a sensitivity of 88.9% and a specificity of 93.7%. The area under the curve was 0.952 (95% confidence interval: 0.917 to 0.986).

The ROC curve shows the accuracy of HbA1c for diagnosing diabetes against FPG ≥7.0 mmol/L among participants without previously diagnosed diabetes (n = 3122) (Fig 1). The optimal cut-off value of HbA1c for diagnosing diabetes was ≥6.5%, with the area under the curve being 0.952 (95% confidence interval: 0.917 to 0.986). Table 2 presents the sensitivity, specificity, positive predictive value, negative predictive value, positive likelihood ratio, and negative likelihood ratio of different HbA1c thresholds for diagnosing diabetes against FPG ≥7.0 mmol/L in participants without history of diabetes. Similarly, the HbA1c cut-off value of ≥6.5% for diagnosing diabetes against FPG ≥7.0 mmol/l yielded a maximal Youden index of 0.825, with the sensitivity and specificity being 88.9% and 93.7%, respectively. As expected, with the increase of cut-off for HbA1c, the sensitivity gradually decreased while specificity increased. The HbA1c cut-off of ≥6.1% (43 mmol/mol) exhibited the highest sensitivity (95.1%) and the cut-off of ≥6.9% (52 mmol/mol) exhibited the highest specificity (97.9%).

**Table 2:** Diagnostic accuracy of diabetes by different cut-offs of HbA1c compared with fasting plasma glucose >7.0 mmol/L among people without previously diagnosed diabetes (n = 3122).

Cut-offs of HbA1c (%)	Sensitivity (%)	Specificity (%)	Youden Index	PPV (%)	NPV (%)	PLR	NLR
`6.1 (mean plus 1 SD)	95.1	78.8	0.738	10.7	99.8	4.5	0.06
6.4	90.1	91.5	0.816	22.0	99.7	10.6	0.11
6.5^*^ (mean plus 2 SD)	88.9	93.7	0.825	27.2	99.7	14.0	0.12
6.6	86.4	95.4	0.818	33.3	99.6	18.8	0.14
6.9 (mean plus 3 SD)	72.8	97.9	0.707	47.6	99.3	34.1	0.28

Abbreviations: NLR, negative likelihood ratio; PPV, positive predictive value; NPV, negative predictive value; PLR, positive likelihood ratio.

Mean plus 1 SD: 1 SD above mean value of HbA1c in participants with normal glucose tolerance.

^*^ The HbA1c cut-off ≥6.5% was recommended by the International Expert Committee with members appointed by the American Diabetes Association, the European Association for the Study of Diabetes, and the International Diabetes Federation. [20]

### 3.3 Agreements of FPG, FSG, and HbA1c in the diagnosis of diabetes

Kappa statistic is used to evaluate the agreement between two diagnostic methods in defining a binary outcome. In the total sample (n = 3547), we defined diabetes using the three methods that integrated self-reported history of diabetes, current use of glucose-lowering drugs and one of the three glycemic measurements, i.e., diabetes was considered to be present if the participant had either the self-reported physician diagnosis of diabetes or use of glucose-lowering drugs or (1) FPG ≥7.0 mmol/l (FPG-defined diabetes), (2) FSG ≥7.0 mmol/l (FSG-defined diabetes), and (3) HbA1c ≥6.5% (HbA1c-defined diabetes). The kappa statistic of the diagnostic agreement was 0.962 between the FPG- and FSG-defined diabetes and 0.812 between the FPG- and HbA1c-defined diabetes (Table 3).

**Table 3:** Diagnostic agreement of diabetes according to different methods in the total sample (n = 3547).

Fasting plasma glucose^*^	Fasting serum glucose^*^		Glycated hemoglobin A1c^*^	
	No diabetes, n (%)	Diabetes, n (%)	No diabetes, n (%)	Diabetes, n (%)
No diabetes, n (%)	3029 (99.6)	12 (0.4)	2848 (93.7)	193(6.3)
Diabetes, n (%)	21 (4.2)	485 (95.8)	9 (1.8)	497 (98.2)
Kappa statistic	0.962^‡^		0.812^‡^	

^*^Diabetes was defined by integrating glucose-lowering drug treatment and medical record of diabetes with either fasting plasma glucose ≥7.0 mmol/L or fasting serum glucose ≥7.0 mmol/L or glycated hemoglobin A1c ≥6.5%, respectively.

^‡^*P*<0.001.

### 3.4 Correlations of FPG, FSG, and HbA1c

**Fig 2:**
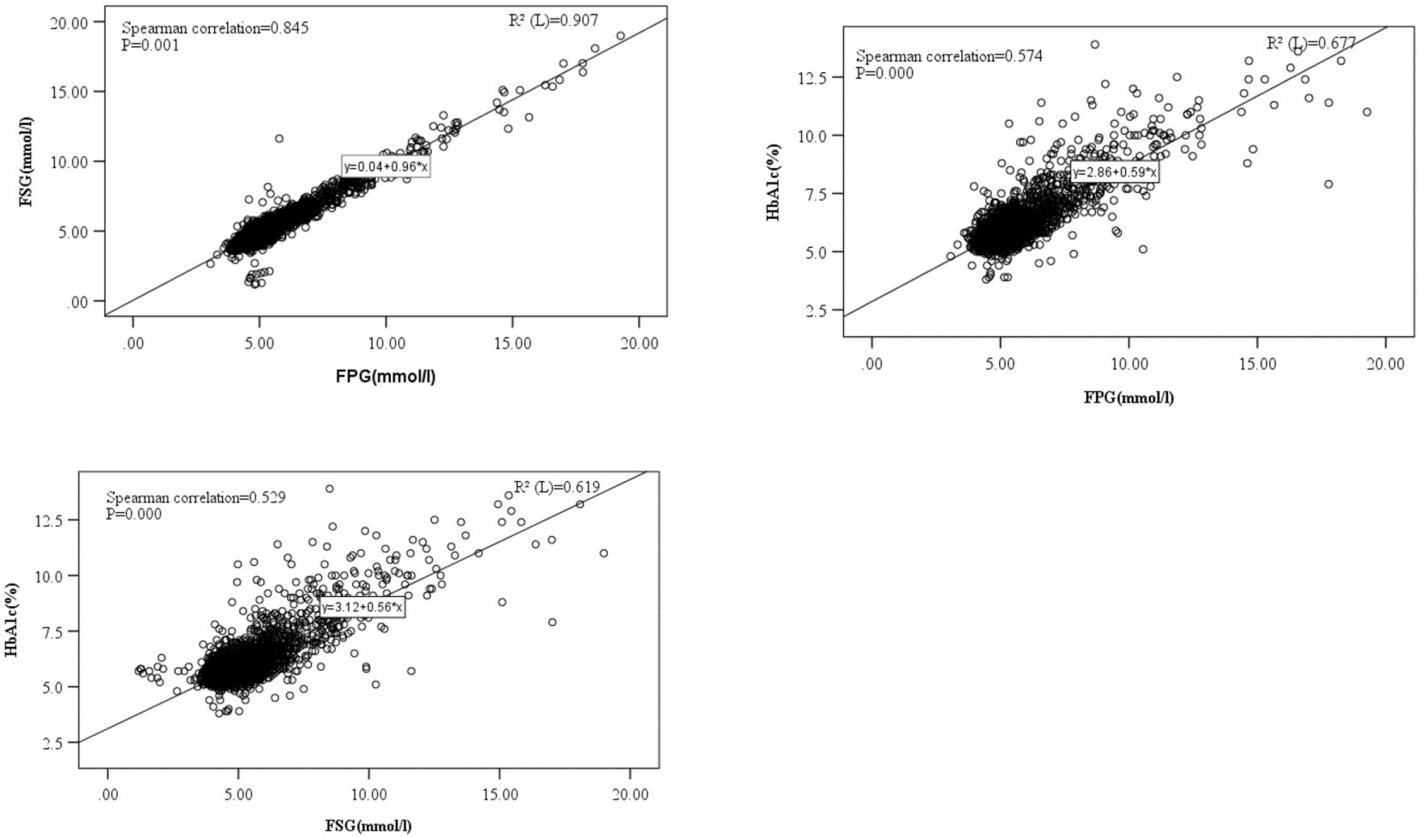
The correlations between fasting plasma glucose, fasting serum glucose, and glycated hemoglobin A1c in the total sample (n = 3547). A. The correlation between fasting plasma glucose and fasting serum glucose. B. The correlation between fasting plasma glucose and glycated hemoglobin. C. The correlation between fasting serum glucose and glycated hemoglobin.

In the total sample (n = 3547), the correlation coefficient between FPG and FSG was 0.845 (Fig 2A), between FPG and HbA1c 0.574 (Fig 2B), and between FSG and HbA1c 0.529 (all P<0.001) (Fig 2C). Among the participants with a known history of diabetes (n = 425), the corresponding correlation coefficients were 0.972, 0.763, and 0.743 (all P<0.001), respectively.

## 4. Discussion

In this population-based cross-sectional study of rural-dwelling Chinese older adults who were aged 61–80 years and free of dementia and disability, we found that (1) the optimal HbA1c cut-off for defining diabetes among individuals without a history of diabetes was ≥6.5%; and (2) FPG (≥7.0 mmol/L), FSG (≥7.0 mmol/L), and HbA1c (≥6.5%) achieved a nearly perfect agreement in defining diabetes.

To the best of our knowledge, this is the first population-based study to investigate the optimal cut-off of HbA1c in defining diabetes among elderly people in China. HbA1c represents the long-term glycemic exposure in red blood cells and reflects the time-weighted average glycemic levels in the past 3–4 months. Therefore, HbA1c is considered a stable measure in diagnosing diabetes. In our study, the optimal HbA1c cut-off for defining diabetes was ≥6.5%, which is consistent with the guidelines of WHO and ADA [15, 20]. However, a previous population-based study in China suggested that the optimal cut-off of HbA1c for defining diabetes was ≥6.3% [21], which was slightly lower than the optimal cut-off from our study. It should be noted that the previous study included Chinese adults over 20 years of age in urban residents. Research has indicated that, as people age, the glycation process increases and the erythropoiesis and clearance of impaired red blood cells decrease, which might lead to the fluctuated level of HbA1c [22, 23]. Thus, the optimal cut-off of HbA1c for diagnosing diabetes might slightly increase with advancing age. Our study suggests that among rural-dwelling Chinese older adults, HbA1c ≥6.5% is the optimal cut-off for defining diabetes, which is in accordance with the recommendation by WHO and ADA. This has significant implications for healthcare practices and diabetes research among rural older adults in China.

Previous studies have compared FPG and FSG in defining diabetes. For instance, data from the Finnish Diabetes Prevention Study showed virtually no difference between FPG and FSG when they were measured instantly; at the FPG level of 7.0 mmol/L the difference was less than 0.2 mmol/L, which was within the range of random variation [13]. In addition, studies have shown that FPG concentrations are slightly higher than FSG concentrations when there is time delay from blood sampling to glycose testing [14, 24], which is consistent with our results: the mean value of FPG was slightly higher than that of FSG (5.4 versus 5.2 mmol/l). This is because the glucose concentrations decrease over time as a result of glycolysis in whole blood ex vivo [25]. These results suggest that there is a strong correlation and an excellent diagnostic agreement between FPG and FSG. FSG appears to be a reliable blood glucose measure for the diagnosis of diabetes if timely measured. Population-based studies have thus far rarely assessed the agreement between FPG and HbA1c for the diagnosis of diabetes in low- and middle-income countries. Our study showed the moderately strong correlation between FPG and HbA1c in the total sample and strong correlation among people with known diabetes, which was in line with the findings from the population-based New Hoorn Study (age 40–65 years) in the Netherlands [26]. The Diabetes Control and Complications Trial also showed that the relationship between FPG and HbA1c might vary depending on the glycemic control of the targeted population with type 1 diabetes [27]. In addition, FPG and HbA1c achieved nearly perfect agreement in defining diabetes in our study, suggesting that HbA1c could be an alternative measure in defining diabetes in the general population of older adults [28].

The major strength of our study is the population-based design engaging in rural-dwelling older adults with the relatively large sample. Our study also has limitations. First, we did not perform the 2-h OGTT, because such a test was not feasible in our large-scale general population setting of older adults. Compared to 2-h OGTT, the use of FPG may lead to under-diagnosis of diabetes. Second, measurements of FPG and HbA1c were slightly delayed due to transportation of blood samples, which might slightly affect the accuracy of these tests in defining diabetes. Third, our study sample consisted of people who were free of dementia and functional disability and derived from a specific rural area of China, which may not be representative of the general elderly population in China. This may not be representative of the general elderly population in China. This should be kept in mind when generalizing our research findings to other elderly populations.

## 5. Conclusion

This population-based study of rural-dwelling older adults in China suggested that optimal HbA1c cut-off value for defining diabetes against FPG ≥7.0 mmol/L was ≥6.5%. Furthermore, there was a nearly perfect agreement among FPG, FSG (≥7.0 mmol/L), and HbA1c (≥6.5%) in diagnosing diabetes in the rural elderly population. These findings have relevant implications for clinical practices and diabetes research among rural older adults in China. Future studies may further evaluate the optimal threshold of HbA1c as a diagnostic tool for diabetes against the gold-standard test (2-hPG) in older adults.
